# Hybrid optimization for ^13^C metabolic flux analysis using systems parametrized by compactification

**DOI:** 10.1186/1752-0509-2-29

**Published:** 2008-03-26

**Authors:** Tae Hoon Yang, Oliver Frick, Elmar Heinzle

**Affiliations:** 1 James Graham Brown Cancer Center & Department of Surgery, 2210 S. Brook St. Rm 342, Belknap Research Building, University of Louisville, Louisville, KY 40208, USA; 2 Biochemical Engineering Institute, Saarland University, Campus A1.5, D-66123 Saarbrücken, Germany

## Abstract

**Background:**

The importance and power of isotope-based metabolic flux analysis and its contribution to understanding the metabolic network is increasingly recognized. Its application is, however, still limited partly due to computational inefficiency. ^13^C metabolic flux analysis aims to compute *in vivo *metabolic fluxes in terms of metabolite balancing extended by carbon isotopomer balances and involves a nonlinear least-squares problem. To solve the problem more efficiently, improved numerical optimization techniques are necessary.

**Results:**

For flux computation, we developed a gradient-based hybrid optimization algorithm. Here, independent flux variables were compactified into [0, 1)-ranged variables using a single transformation rule. The compactified parameters could be discriminated between non-identifiable and identifiable variables after model linearization. The developed hybrid algorithm was applied to the central metabolism of *Bacillus subtilis *with only succinate and glutamate as carbon sources. This creates difficulties caused by symmetry of succinate leading to limited introduction of ^13^C labeling information into the system. The algorithm was found to be superior to its parent algorithms and to global optimization methods both in accuracy and speed. The hybrid optimization with tolerance adjustment quickly converged to the minimum with close to zero deviation and exactly re-estimated flux variables. In the metabolic network studied, some fluxes were found to be either non-identifiable or nonlinearly correlated. The non-identifiable fluxes could correctly be predicted *a priori *using the model identification method applied, whereas the nonlinear flux correlation was revealed only by identification runs using different starting values *a posteriori*.

**Conclusion:**

This fast, robust and accurate optimization method is useful for high-throughput metabolic flux analysis, *a posteriori *identification of possible parameter correlations, and also for Monte Carlo simulations to obtain statistical qualities for flux estimates. In this way, it contributes to future quantitative studies of central metabolic networks in the framework of systems biology.

## Background

In recent years, metabolic flux analysis (MFA) has become an important tool for quantifying metabolic pathways which is essential for in-depth understanding of biological systems. Among the developed tools, ^13^C flux analysis utilizing ^13^C labeling patterns of metabolic products that result from feeding ^13^C-labeled substrates provides detailed information about intracellular pathway fluxes *in vivo *[1-3]

^13^C-based MFA requires carbon flux modeling through the metabolic network, which describes the mathematical relationship between unknown fluxes and the available measurement data set. It requires modeling two connected equation systems, which describe reaction stoichiometry between metabolites and between carbon isotopomers, respectively. Using the model, fluxes can be computed from the measurements by solving a nonlinear least-squares problem (NLSP). The stoichiometric network is a linear equation system of material balances given for the reactions between metabolites, i.e., **S·ν **= **0 **with **S **denoting the stoichiometric matrix and **ν **the fluxes. The fluxes consist of non-measured intracellular fluxes **ν**_u _and measured effluxes **ν**_m_, i.e., **ν **= (**ν**_u_, **ν**_m_)^*T*^. Typically, realistic models are underdetermined, that is, the rank of **S **is smaller than the number of entries in **ν**_u _[1]. This difference between *rank*(**S**) and the number of entries of **ν**_u _equals the number of fluxes that have to be chosen as the design parameters **Θ **(independent flux variables) and are required when parametrizing the network such that **ν **= **F**_flux_(**Θ**).

Typically, modeling carbon isotopomer networks involves an equation system containing a few to several hundred variables to balance reactions between isotopomers [1,3,4]. The equation system of carbon isotopomer reactions is an implicit function **F**_carbon_(**x**, **ν**) = **0**, bilinear but square with respect to carbon isotopomer fractions (**x**) and nonlinear with respect to fluxes, where **x **consists of non-measured **x**_u _and measured **x**_m_. Among the available tools, the cumomer concept developed by Wiechert *et al*. (1999) provides explicit solutions for carbon isotopomer fractions (**x**) by transforming the bilinear system into a cascade of linear systems [5]. In addition to this, the explicit partial derivatives of the cumomer network with respect to fluxes are obtainable, which is useful for gradient-based optimization algorithms. When ^13^C labeling information is applied, intracellular fluxes are determined by means of numerical optimization that seeks a constrained minimum of independent flux variables (**Θ**) to an objective function, e.g.,

$$\underset{\Theta \in {ℜ}^{m}}{\min}f(\Theta )=\frac{1}{2}{\left(\eta -F(\Theta )\right)}^{T}\cdot \Sigma_{\eta }^{-1}\cdot \left(\eta -F(\Theta )\right)\text{subject to }\nu \text{(}\Theta \text{)}\geq 0.$$

Here *f*(**Θ**) denotes the objective function to be minimized with respect to **Θ **and F (**Θ**) signifies the model function corresponding to the measured data set **η **= (η_1_, η_2_,..., η_*n*_)^*T *^consisting of measured ^13^C labeling data (**x**_m_). Such data are typically received as mass isotopomer distributions measured by mass spectrometry (MS) and/or fractional carbon labeling measured by nuclear magnetic resonance (NMR) techniques, and effluxes (**ν**_m_). The measurement error **ε **is typically assumed to have a normal distribution such that **ε **∈ ***N***(**0**, **Σ**_*η*_), where **Σ**_*η *_is the covariance matrix of measurements.

For ^13^C-based MFA, the applied algorithms for numerical flux estimation are mainly gradient-based local optimization [6,7] or gradient-free global optimization [8-11] such as simulated annealing (SA) or genetic algorithms (GAs). Also, a hybrid technique of global-local optimization has been applied [12]. Such algorithms are described in detail elsewhere [13-15]. The stochastic global optimization methods can be inefficient due to the time required to obtain the so-called asymptotic convergence or reachability in high dimensional parameter spaces [11,16-21]. Moreover, such algorithms of random nature may fail to find the global solution unless the number of samplings tends to infinity, which is practically impossible [21,22]. In comparison, the gradient-based local optimizations have a much higher convergence speed, but the solution quality depends heavily on starting points [14,19]. Further to this, reaching the global optimum is ensured only for convex problems, whereas it is nontrivial to determine the convexity of general nonlinear problems with nonlinear constraints [23]. Thus, one may obtain solutions that are not necessarily global [14] and that might vary depending on starting points.

In this regard, a robust method is highly desirable which guarantees a convergence with speed and accuracy for the nonlinear flux estimation problem. In the present work, we developed a method for efficient parametrization of metabolic network by compactification. Additionally, a mathematical method is suggested for model identification that solves *a priori *flux identifiability problems regarding ^13^C labeling experiments in terms of model linearization. On this basis, an optimization algorithm was developed that hybridizes two gradient-based optimization tools. The developed approaches were evaluated using the central metabolism of *Bacillus subtilis *with succinate and glutamate feeding as the only carbon sources. We examined whether global flux solutions are obtainable using the gradient-based deterministic algorithm.

## Results

In this section, we describe the mathematical and computational procedures developed and their application to the nonlinear problem of metabolic flux estimation in a realistic metabolic network of *Bacillus subtilis*. The model represents an inherent difficulty arising from only succinate and glutamate feeding as carbon sources. Succinate is a symmetric molecule having only two distinguishable carbon atoms. On the other hand, glutamate is also converted into succinate. Due to this, the information that is introduced by the ^13^C-labels of the substrates is severely limited.

### Parametrization of stoichiometric network

To formulate a non-linear least squares problem (NLSP), the system of interest has to be parametrized. The stoichiometric network, which is a rank-deficient linear system (**S·ν **= **0**), was parametrized using the following three steps.

#### Step (i)

The stoichiometric matrix **S **is transformed into its reduced row echelon form **S**_RRE _using Gauss-Jordan elimination with partial pivoting. Subsequently, each row and column of **S**_RRE _is analyzed to identify the dependent and independent variables. The first non-zero element in each row of **S**_RRE _that is always 1 is called 'leading 1'. If the *i*^th ^column of **S**_RRE _contains only zeros and one leading 1, then the *i*^th ^element of **ν **is dependent, otherwise it is independent. The number of fluxes selected as independent equals the degrees of freedom in the stoichiometric network, that is, the difference between the number of variables and the rank of **S**. Once this step has been completed, we will see that certain intracellular fluxes and all effluxes (**ν**_m_) are chosen as independent.

#### Step (ii)

Physiologically, metabolic fluxes are constrained such that **0 **≤ *ν *< ∞, if reversible reactions are considered as two independent reactions. For the effluxes, the corresponding measurements are available. Hence, the effluxes can be bounded, e.g., mean value ± $\sqrt{\chi_{\text{1,}\varphi }^{\text{2}}}$ × standard deviation, where $\chi_{\text{1,}\varphi }^{\text{2}}$ denotes the inverse of *χ*^2^-cumulative distribution function at a certain confidence level of *φ*, and the resulting range covers 100 × *φ *% of possible experimental observations.

The intracellular fluxes selected as independent can be compactified using a single rule such that:

$$\begin{array}{ccc} \phi_{i}=\frac{\nu_{i}}{\alpha +\nu_{i}} & \text{with} & 0\leq \nu_{i}<\infty \wedge \alpha >0\Rightarrow \phi_{i}\in [0,\;1). \end{array}$$

The above compactified flux variables *ϕ*, the [0, 1)-fluxes, are a bijection of [0, ∞) domain onto [0, 1) range: if *ν*_*i *_→ 0, *ϕ*_*i *_→ 0 and if *ν*_*i *_→ ∞, *ϕ*_*i *_→ 1 for a certain real positive number of the parameter scaling constant *α*. These [0, 1)-fluxes can potentially elevate the output sensitivity and, thus, the convergence speed. The output sensitivities of a carbon flux system with respect to fluxes and [0, 1)-fluxes are ∂**x**_m_/∂**ν **and ∂**x**_m_/∂**ϕ**, respectively, where the latter equals (∂**x**_m_/∂**ν**)·(∂**ν**/∂**ϕ**) by the chain rule. Differentiating *ν*_*i *_with respect to *ϕ*_*i *_results in

$$\frac{d\nu_{i}}{d\phi_{i}}=\frac{\alpha }{{\left(1-\phi_{i}\right)}^{2}}$$

from (2). If *α *> (1 - *ϕ*_*i*_)^2 ^holds and, thus, *dν*_*i*_/*dϕ*_*i *_> 1, the sensitivity given by ∂**x**_m_/∂**ϕ **= (∂**x**_m_/∂**ν**)·(∂**ν**/∂**ϕ**) increases. Particularly, a higher sensitivity can always be obtained by setting *α *≥ 1 due to finite values of fluxes, that is, 0 ≤ *ν*_*i *_< ∞ or 0 ≤ *ϕ*_*i *_< 1. Hence, setting the parameter scaling constant *α *≥ 1 is more preferable for numerical optimization than *α *> 0. Moreover, the mapping such as (2) has proven to yield an extremely low curvature of ^13^C labeling in the parameter space and is advantageous for model linearization [24]. The constant *α *can be adjusted during the optimization. This is described in detail in the Appendix.

#### Step (iii)

By symbolically solving the equation system consisting of stoichiometric balances and flux constraints such as (2) for the dependent fluxes (**ν**_depend_), we get explicit expressions for all dependent fluxes such that **ν**_depend _= **F**_flux_(**Θ**) with **Θ **= (*ϕ*_1_, *ϕ*_2_,..., *ν*_m1_, *ν*_m2_,...)^*T*^.

### Flux identifiability by model linearization

One important question to be answered prior to the numerical flux computation is the *a priori *parameter identifiability. To this end, the so-called Buchberger's algorithm [25] can be applied to compute the Gröbner basis [26]. However, this polynomial algebraic method can be very time consuming and, thus, is restricted to small systems, corresponding to the fact that the Gröbner basis can be extremely large for a metabolic carbon labeling system [27,28].

In previous works [28,29], the Jacobian matrix of the measurement model has been utilized for model identification: the matrix determinant or rank were applied to the flux identifiability analysis in carbon labeling systems. However, these approaches do not tell how to sort out identifiable flux variables in case not all fluxes are identifiable.

As an alternative, the first order optimality condition and null space analysis can be applied to model identification. For this purpose, the linear algebra approach is now extended to answer the question of the *a priori *flux identifiability problem regarding ^13^C isotopomer analysis, which begins with model linearization. At the solution of the nonlinear problem (1), the gradient ∇*f *is expected to be 0 by the first order optimality condition, as in:

$$\nabla f(\hat{\Theta })=J{\left(\hat{\Theta }\right)}^{T}\cdot \Sigma_{\eta }^{-1}\cdot \left(F(\hat{\Theta })-\eta \right)=0.$$

Here, **J**($\hat{\Theta }$) represents the Jacobian matrix that equals ∂F/∂**Θ **evaluated at $\hat{\Theta }$. Assuming that the parameter estimate $\hat{\Theta }$ is close to its true value $\hat{\Theta }$, the above equation can linearly be approximated in the neighborhood of $\hat{\Theta }$ by the first-order Taylor series expansion such that:

$$J{\left(\hat{\Theta }\right)}^{T}\cdot \Sigma_{\eta }^{-1}\cdot \left(F(\hat{\Theta })+J(\hat{\Theta })\cdot \Delta \hat{\Theta }-\eta \right)=0.$$

Solving the above equation for $\Delta \hat{\Theta }$ gives the particular solution of (1) as follows:

$$\Delta \hat{\Theta }={\left(J{\left(\hat{\Theta }\right)}^{T}\cdot \Sigma_{\eta }^{-1}\cdot J(\hat{\Theta })\right)}^{-1}\cdot J{\left(\hat{\Theta }\right)}^{T}\cdot \Sigma_{\eta }^{-1}\cdot \left(\eta -F(\hat{\Theta })\right).$$

Assuming that the above linearization is a good approximation in the vicinity of solution, the *a priori *identification problem can be answered using linear system theory, similarly as applied for stoichiometric metabolite balancing [30]. The theory is now extended to the linearized carbon labeling system: the general solution of (1) can be formulated as a linear combination of the particular solution (6) with its homogeneous part, i.e.,

$$\Delta \hat{\Theta }={\left(J{\left(\hat{\Theta }\right)}^{T}\cdot \Sigma_{\eta }^{-1}\cdot J(\hat{\Theta })\right)}^{-1}\cdot J{\left(\hat{\Theta }\right)}^{T}\cdot \Sigma_{\eta }^{-1}\cdot \left(\eta -F(\hat{\Theta })\right)+null(J(\hat{\Theta }))\cdot \beta .$$

Here, *null*(**J**($\hat{\Theta }$)) is the null space of **J**($\hat{\Theta }$) and **β **is a vector containing arbitrary non-zero values. The *i*^th ^element of the search step $\Delta \hat{\Theta }$ whose corresponding row of *null*(**J**($\hat{\Theta }$)) consists of zeros can be determined uniquely from the measurement **η**. Thus, the unique solution of the corresponding *i*^th ^design parameter can be computed numerically.

### Hybrid optimization algorithm

Due to the parametrization introduced above, the flux estimation problem (1) can now be formulated such that:

$$\begin{array}{ccc} \underset{\Theta }{\min}f(\Theta ) & \text{subject to} & \{\begin{array}{c} \nu_{\text{depend}}(\Theta )\geq 0\\ 0\leq \phi_{i}<1\\ \max(0,-\sqrt{\chi_{\text{1,}\varphi }^{\text{2}}}\sigma_{\text{m}})\leq \nu_{\text{m}}\leq \sqrt{\chi_{\text{1,}\varphi }^{\text{2}}}\sigma_{\text{m}} \end{array} \end{array}\text{,}$$

where *f*(**Θ**) denotes the objective function specified in (1) and **Θ **= (*ϕ*_1_, *ϕ*_2_,...,*ϕ*_*n*_, *ν*_m1_, *ν*_m2_,...)^*T *^the design parameter vector consisting of the [0, 1)-fluxes *ϕ*_*i*_. The effluxes *ν*_m _comprise substrate uptake as well as product and biomass formation with known standard deviation **σ**_*m*_, and **ν**_depend _corresponds to all unknown intracellular fluxes.

**Figure 1:**
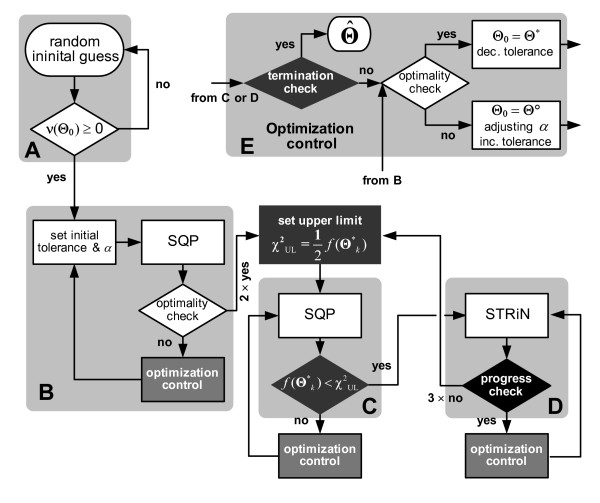
Developed hybrid optimization algorithm with tolerance adjustment consisting of the features: initialization within the feasible region (A); initial optimization using the SQP (B); interactive hybrid process using SQP (C); STRiN (D); and optimization control algorithm (E). *f*(Θ*_k_): objective function value at the current local minimizer Θ*_k_; *χ*^2^_UL_: upper limit of f(Θ*_k_) to invoke STRiN (if *f*Θ*_k_) <*χ*^2^_UL_; *α*: parameter scaling constant; Θ_0_: initial guess; Θ*: local minimizer from a successful sub-optimization; Θ°: iterate recorded for the smallest function value up to the current optimization trial; $\hat{\Theta }$: ultimate minimizer.

To solve the above constrained NLSP, we developed a logical algorithm (Figure 1) that interactively hybridizes two gradient-based local optimization methods, that is, the sequential quadratic programming (SQP) [31] and the subspace trust-region method based on the interior-reflective Newton method (STRiN) [32]. The developed method performs a series of sub-optimization trials by interactively switching between SQP and STRiN using the following features.

#### Analytical gradient and Hessian

Typically, a gradient-based optimization problem is solved more accurately and with higher efficiency when analytical gradients are provided compared to numerical gradients, which are calculated by finite-difference approximation. By providing the analytical Jacobian matrix consisting of ∂**x**_m_/∂**ϕ **and ∂**ν**_m_/∂**Θ**, the gradient of the objective function formulated in (A.1) of the Appendix can be calculated. The analytical Hessian (A.2) is calculated when STRiN is used. In comparison to this, SQP updates the Hessian using the Broyden-Fletcher-Goldfarb-Shanno (BFGS) formula based on linearization of the gradient [14].

#### Adjustment of tolerance and parameter scaling constant

Usually, the tolerances placed on parameters or on the objective function which are utilized as termination criteria for numerical optimization cannot clearly be defined in advance. They depend on the errors associated with user-supplied data as well as output sensitivities. Thus, tolerances are set rather empirically. Here, we suggest a method to adjust tolerance values and find a proper value by repeatedly restarting optimization trials. The tolerance adjustment by the restart is implemented in the developed algorithm as shown in Figure 1-E. Due to this, the complete process consists of a series of sub-optimization trials. At each *k*^th ^trials, tolerance values are adjusted.

Further to this, the parameter scaling constant *α *is also adjusted during the optimization. As mentioned in the previous section, *α *≥ 1 is the preferable choice due to the finite values of metabolic fluxes. In practice, *α *is regulated to render the model's Jacobian matrix into better condition. This is obviously advantageous for the gradient-based method, which typically involves matrix inversion to compute the search direction as shown by (6). The exact procedure how to adjust tolerance and the parameter scaling constant is described in the Appendix.

#### Hybridization

SQP represents the state-of-the-art for solving constrained nonlinear optimization problems. By introducing the Lagrangian function, SQP solves a series of quadratic programming sub-problems, each involving the minimization of a quadratic approximation of the objective function subject to a linear approximation of the constraints. It shows its strength when solving problems with significant nonlinearity and from remote starting points [14,33].

STRiN solves a nonlinear large problem by linear approximation and the method of preconditioned conjugate gradients. This algorithm is based on the trust-region algorithm, is computationally inexpensive, and provides rapid convergence in the vicinity of the solution. A downside of STRiN is that it accepts any direction of negative curvature, even when this direction gives an insignificant reduction in the objective function [14]

The key concept of the developed algorithm is hybridizing the merits of these two optimization algorithms, i.e., the loose starting-point-dependency of SQP and the convergence speed of STRiN in the solution vicinity. During the optimization trails with the tolerance adjustment, we get a feasible point that may be a more suitable initial guess for the next trial. Accordingly, we initiate a few trials under "relaxed" tolerance conditions using a robust algorithm that is less dependent on the quality of the initial points. Subsequently, the local minima obtained can be tested by another algorithm whether it gives a significant improvement with a rapid convergence.

In this context, the hybridization is carried out as shown in the flow chart in Figure 1. The algorithm is forced to initiate within the feasible region by generating an arbitrary starting point **Θ**_0 _subject to the inequality constraint **ν**_depend_(**Θ**_0_) ≥ **0 **(Figure 1-A). This can be done by generating random numbers of design parameters within their bounds given by (8). For cases in which the random generation is expensive, e.g., when a design parameter has a very narrow range that satisfies the constraints, a deterministic method might be employed to get a starting point in the physically possible region. This is discussed in the Appendix.

As soon as a feasible set of initial values is obtained, the optimization starts using the SQP algorithm under relaxed tolerance criteria (Figure 1-B). After a few successful trials, an upper limit (*χ*^2^_UL_) that schedules the initiation of STRiN is placed, e.g., half of the current objective function value, *χ*^2^_UL _= 1/2 *f*(Θ*_k_), where Θ*_k _is the local minimizer isolated from the *k*^th ^sub-optimization trial. Afterwards, if the objective value *f*(Θ*_k_) resulting from the current SQP trial (Figure 1-C) is smaller than *χ*^2^_UL_, the STRiN optimization is activated (Figure 1-D); otherwise, the SQP is repeated. During the STRiN optimization, the progress of trials is monitored to prevent the non-reducing problem associated with the STRiN algorithm mentioned above. As a criterion of the progress check, the relative improvement of |*f*(Θ*_k_) - *f*(Θ*_k-1_)|/*f*(Θ*_k_) can be measured at each STRiN termination. If the improvement is insignificant, i.e., less than a user-specified value (e.g., 0.05), the STRiN receives penalty. In case a few successive trials show insignificant improvement, the algorithm sets a new upperlimit *χ*^2^_UL _and returns to the SQP optimization. For the restart, the tolerance of the previous SQP trial is reutilized. This prevents too large changes in tolerance, which can be caused by the STRiN trials with insignificant improvement.

As a termination criterion of the hybrid optimization, the changes in local optima are measured (Figure 1-C), e.g., the absolute value of the slope resulting from the linear regression of y = (*f*(Θ*_k-4_), *f*(Θ*_k-3_),...*f*(Θ*_k_))^*T *^with respect to x = (1, 2,..., 5)^*T*^. If the absolute slope is less than a user-specified small value for a few further trials, the optimization can be terminated ultimately.

### Application to *Bacillus subtilis *metabolic network

**Figure 2:**
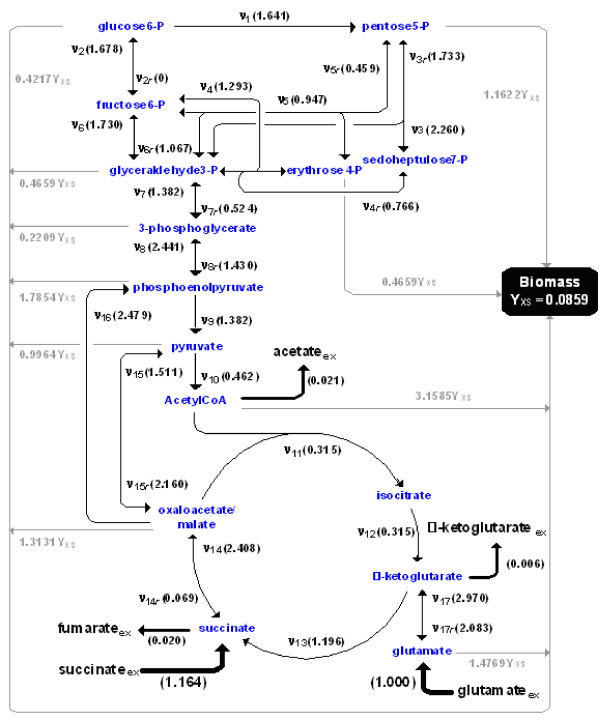
Metabolic network of the central metabolism of *Bacillus subtilis *utilizing glutamate and succinate as co-substrates. All flux values denoted in parentheses were generated by obeying the given stoichiometry. Effluxes and biomass yield were measured experimentally. The symbol '**ν**' indicates the flux, the subscript '***r***' the reverse flux of the bidirectional flux pair, the subscript '**ex**' extracellular pools of substrates and products and Y_*XS *_the biomass yield in g(biomass)/mmol(glutamate). All flux values are normalized by the glutamate uptake rate.

To evaluate the developed methods, a metabolic network of the wild type *B*.*subtilis *was constructed as shown in Figure 2 based on the Kyoto Encyclopedia of Genes and Genomes (KEGG) database specified for the strain. The network is composed of catabolic reactions of the central metabolism incorporating glycolysis/gluconeogenesis, pentose phosphate pathway, TCA cycle, C3/C4 inter-conversion and anabolic reactions. All effluxes including the biomass yield Y_XS _in Figure 2 were assumed to be measured experimentally from a batch-cultivation on succinate and glutamate as carbon sources. All flux values specified in Figure 2 are generated from arbitrary values of **Θ**_default _and normalized by the glutamate uptake rate. Note that each anabolic flux given in Figure 2 is the product of Y_XS _(biomass production [g_DW _L^-1 ^h^-1^] normalized by glutamate uptake [mM h^-1^]) and a value that specifies the precursor requirement for growth (mmol precursor per g biomass) adopted from literature data [34]. For bidirectional reactions, the fluxes in the gluconeogenetic direction were declared as forward.

#### Parametrization

The metabolic network in Figure 2 has 27 intracellular fluxes (10 bidirectional and 17 unidirectional fluxes), 5 effluxes, and 10 anabolic fluxes expressed in terms of Y_*XS*_. For 15 intracellular metabolites defined in the network, 15 flux balances were set up that are linearly independent of each other (Appendix). The stoichiometric matrix **S **was obtained by symbolic differentiation of the balances with respect to the whole fluxes including Y_XS_. When considering that the flux of glutamate uptake is unity due to the normalization, the network owns 17 degrees of freedom. Accordingly, 12 intracellular fluxes *ν*_2*r*_, *ν*_3*r*_, *ν*_4*r*_, *ν*_5*r*_, *ν*_6*r*_, *ν*_7*r*_, *ν*_8*r*_, *ν*_14_, *ν*_14*r*_, *ν*_15*r*_, *ν*_16_, *ν*_17*r*_, 4 effluxes, and Y_XS _were recognized as independent variables from the reduced row echelon form **S**_RRE _of **S**. These independent intracellular fluxes were transformed into [0, 1)-fluxes as given by (2). Note that the counterpart of a backward flux of a bidirectional reaction can also be selected as an independent variable. This just depends on how to arrange the entries in the flux vector. For instance, when *ν*_2*r *_is followed by *ν*_2 _in the flux vector, *ν*_2 _will be recognized as an independent variable instead of *ν*_2*r*_.

Solving the equation system of the flux balances (Appendix) and the [0, 1)-flux equations for the dependent fluxes gives their explicit analytical expressions with respect to design parameters, i.e., *ν*_*i *_= *n*_flux_(**Θ**) with 19 design parameters of **Θ **= (*ϕ*_2*r*_, *ϕ*_3*r*_, *ϕ*_4*r*_, *ϕ*_5*r*_, *ϕ*_6*r*_, *ϕ*_7*r*_, *ϕ*_8*r*_, *ϕ*_14_, *ϕ*_14*r*_, *ϕ*_15*r*_, *ϕ*_16_, *ϕ*_17*r*_, succinate_ex_, acetate_ex_, *α*-ketoglutarate_ex_, fumarate_ex_, Y_XS_, *p*CO_2,1_, *p*CO_2,2_)^*T*^. The parameters *p*CO_2,1 _and *p*CO_2,2 _estimate the CO_2 _labeling pattern for each ^13^C labeling experiment: CO_2 _has only one carbon and is incorporated into other metabolites by carboxylation. Thus, its labeling state can simply be determined from other ^13^C labeling data as an additional parameter of NLSP in case it is not measured.

For flux re-estimation studies, the default values of the design parameter were **Θ**_default _= (0, 0.6341, 0.4337, 0.3148, 0.5162, 0.3436, 0.5884, 0.7066, 0.0643, 0.6835, 0.7125, 0.6756, 1.164, 0.021, 0.006, 0.02, 0.0859, 0.5601, 0.5601) at *α *= 1, which results in the flux values given in Figure 2.

#### Model identification

For the identifiability studies, the main question was whether **Θ **has one unique solution set that can be estimated from the available mass isotopomer measurements in terms of the gradient-based optimization. The effluxes (succinate_ex_, acetate_ex_, *α*-ketoglutarate_ex_, fumarate_ex_) and Y_XS _are excluded for the identifiability analysis because they can be estimated as long as the corresponding measurements are available. To examine various flux scenarios, 200 stochastic simulations were performed for each of the 630 possible ^13^C-experimental designs. To this end, uniform random numbers between 0 and 0.95 were created for **Θ**. At each simulation the null space of **J**(**Θ**) was calculated.

According to the stochastic simulations, none of the designs had an empty null space and the entry of *null*(**J**(**Θ**)) corresponding to *ϕ*_2*r *_was always non-zero disregarding the flux state and designs. Most designs, including the design considered here, yielded an identical null space of:

*null*(**J**(**Θ**)) = (1 0 0 0 0 0 0 0 0 0 0 0 0 0)^*T*^.

Accordingly, the design parameter *ϕ*_2*r *_is not expected to have a unique solution, i.e., it has infinitively many solutions. Except *ν*_2 _and *ν*_2*r*_, other fluxes were not functions of *ϕ*_2*r*_. This means that the bidirectional glucose 6-P isomerase cannot be determined from any ^13^C substrates or dual substrate combinations. Only the net flux of the reaction can be calculated from the stoichiometry. Therefore, one can either regard the glucose 6-P isomerase as a unidirectional reaction or set *ϕ*_2 _as an arbitrary constant. This renders the carbon flux network to have an empty null space of **J**(**Θ**), i.e., full rank for **J**(**Θ**), and all other design parameters are theoretically expected to have unique solutions. This theoretical expectation is examined later using the developed algorithm.

#### Hybrid optimization with tolerance adjustment

To evaluate the efficiency of the developed optimization algorithm, the flux values calculated in advance were re-estimated numerically from the carbon mass isotopomer distributions (MDVs) of output metabolites matching the default flux values (**ν**_default_). To this end, the default flux values, depicted in Figure 2, were calculated by the flux function **F**_flux_(**Θ**_default_) obtained by parametrization. Subsequently, the ^13^C labeling data of output metabolites were calculated from **ν**_default _and **Θ**_default_. The default fluxes were re-estimated by solving the constrained NLSP (8) whose inputs (**η**) were the effluxes, Y_XS_, and the MDVs.

**Figure 3:**
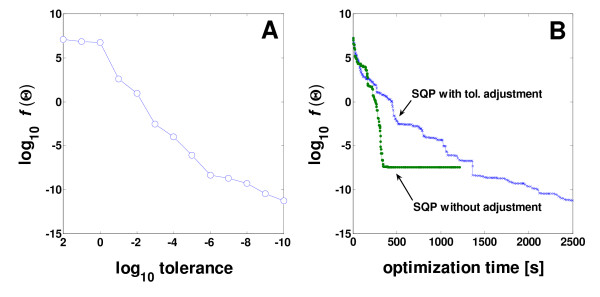
Decrease of the objective function at each termination of SQP sub-optimization using tolerance adjustment (**A**) and its comparison with SQP carried out at a constant tolerance during optimization (**B**).

Prior to testing the hybrid optimization with tolerance adjustment (HATA), we examined whether the tolerance adjustment is beneficial for optimization. This was checked by performing the SQP optimization by providing the gradients for the objective function and for the flux inequality constraints (∇*c *= (-∂**ν**_depend_/∂**Θ**)^*T*^) analytically. As shown in Figure 3-A, the objective function value *f*(Θ*) of each optimization trial decreased with respect to tolerance adjusted at each optimization restart. It was observed that *f*(Θ*_k_) <*f*(Θ*_k-1_) always holds when starting the *k*^th ^trial from the (*k *- 1)^th ^local minimizer Θ*_k-1_. Restarting the failed (*k *- 1)^th ^trial from the feasible iterate **Θ**° recorded for the smallest function value up to the current trial and increasing the tolerance was observed to give the same result, i.e., *f*(Θ*_k_) <*f*(**Θ**°). The efficiency of tolerance adjustment was further compared to the SQP optimization carried out at a constant tolerance of 1 × 10^-20 ^(Figure 3-B). The SQP using the tolerance adjustment was observed to be more efficient in accuracy but more time-consuming than the case without adjusting. The SQP without adjustment reached a local minimum of around 10^-7 ^much more rapidly but did not result in any further improvement, whereas the SQP with adjustment made slower but continuous progression. This gives an idea that the tolerance adjustment strategy might be useful to escape from possible local stationary regions and to achieve a lower minimum.

**Figure 4:**
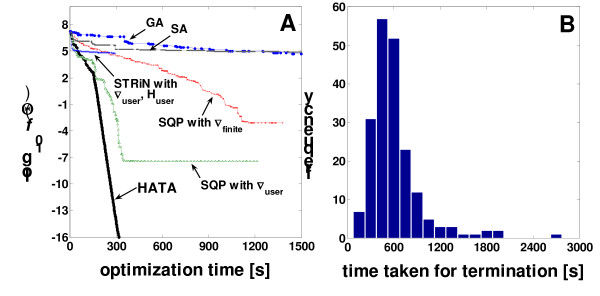
Comparison of the hybrid optimization with tolerance adjustment (HATA) with its parent algorithms of the SQP with user-supplied analytical gradient (SQP ∇_user_) and the STRiN with user-supplied analytical gradient and Hessian (STRiN ∇_user _**H**_user_) as well as with other algorithms, such as SQP with numerical gradient by finite differentiation (SQP ∇_finite_), genetic algorithm (GA), and simulated annealing (SA) (**A**). All algorithms were initiated from an identical starting point. The objective function value at the *i*^th ^iterate is registered only if *f*(**Θ**_*i*_) ≤ *f*(**Θ**_*i*-1_). Time efficiency of the HATA represented by histogram plot of time taken for termination of 200 runs of optimization using different starting points (**B**).

Using the tolerance adjustment, the hybrid algorithm consisting of SQP and STRiN (HATA, Figure 1) was compared with its parent algorithms and two global optimization methods. All optimizations except the genetic algorithm (GA) were initiated from an identical starting point for the numerical flux re-estimation. The GA applied does not need an external initial value set. At each initiation of the HATA trials, *α *was updated by choosing an integer between 1 and 10 that yields the best-conditioned Jacobian matrix of the model as mentioned previously. As shown in Figure 4-A, HATA accomplished the re-estimation with the best efficiency regarding its accuracy and speed. It took about 300 seconds until the objective function became 10^-16^. The SQP optimization with analytical gradient (SQP ∇_user_) yielded the next satisfactory result. In comparison, the SQP optimization using the numerical gradient obtained by the finite difference (SQP ∇_finite_) resulted in a poorer progress and was much slower and less accurate than HATA or SQP ∇_user_. During the optimization using SQP ∇_finite_, we observed a discrepancy between the gradient obtained by the finite difference and the analytical approach (A.1). The inaccuracy of the finite difference seemed to cause the poorer result of SQP ∇_finite_.

The worst algorithm among the local methods was the STRiN using analytical gradient and Hessian (STRiN ∇_user _**H**_user_). It was rapid at the beginning but improved the objective only from 10^7 ^to 10^5^. Afterwards, the optimization crashed with the carbon labeling system becoming completely singular. As previously mentioned, this seems to be due to the STRiN limitation of accepting any direction of negative curvature even if it does not give significant reduction in the objective function. Also, the global optimization methods tested, which were the GA and the SA, were notably time-inefficient compared to the local methods. For 1500 seconds of optimization time, the objective function was very slowly decreased by two orders of magnitude only. SA decreased *f*(**Θ**) only to 1 × 10^3 ^after 5.1 hours and GA to 2 × 10^2 ^after 6.7 hours. Thus, global optimization is expected to demand much greater time to get the equivalent result as HATA.

#### HATA optimization from different starting points

The flux re-estimation was also performed from 200 different starting points created randomly and by simultaneously generating uniform random numbers for the [0, 1)-flux *ϕ*_2*r *_= *ν*_2*r*_/(*α *+ *ν*_2*r*_). This was to examine whether the quality and speed of convergence has any starting-point-dependency and whether *ϕ*_2*r*_, which *a priori *expected to have infinite solutions, has any influence on optimization results.

All 200 optimization runs using the hybrid algorithm were terminated successfully. On average, HATA required 5.3 ± 1.6 min to converge to a weighted objective function value smaller than 1 × 10^-10^, which equals the non-weighted sum of squares less than 2 × 10^-18^. As shown in Figure 4-A, 99.5 % of the flux re-estimations were completed within 10 min and about 76 % in less than 6 min.

**Figure 5:**
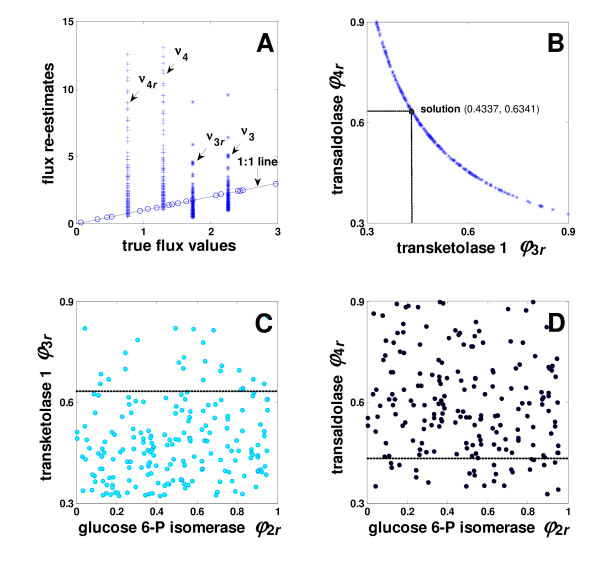
Flux re-estimation initiated from 200 random starting points. The fluxes which were successfully re-estimated lie on the 1:1 line when plotted against true flux values (A). Correlation analysis by plotting the transaldolase (TA) [0, 1)-flux re-estimates (*ϕ*_4*r*_) versus those of transketolase 1 (TK1) (*ϕ*_3*r*_) (B) and the [0, 1)-flux re-estimate of TK1 (C) as well as of TA (D) versus *ϕ*_2*r *_of glucose 6-P isomerase. The dotted line indicates the solution of *ϕ*_3*r *_= 0.4337 and *ϕ*_3*r *_= 0.6341, respectively.

Further to this, the flux re-estimates resulting from the 200 random simulations were compared with the true flux solutions calculated from **Θ**_default _in advance. As shown in Figure 5-A, all fluxes except the flux pairs of transketolase 1 (TK1; *ν*_3_, *ν*_3*r*_) and transaldolase (TA; *ν*_4_, *ν*_4*r*_) could be re-estimated correctly. When plotting the flux re-estimates versus the true flux values given in Figure 5, the data points except *ν*_3_, *ν*_3*r*_, *ν*_4_, and *ν*_4*r *_lie exactly on a line with a slope of 1 (1:1 line). In comparison to this, only the net fluxes of the TK1 (*ν*_3 _- *ν*_3*r*_) and TA (*ν*_4 _- *ν*_4*r*_) reactions could be recalculated properly: the net fluxes could be calculated from stoichiometric relations with other flux re-estimates. The same was observed for the net flux of glucose 6-P isomerase, of which [0, 1)-flux *ϕ*_2*r *_had an arbitrary value at each simulation run.

By the null space investigation (9) based on (7), the design parameters *ϕ*_3*r *_and *ϕ*_4*r *_were not expected to be correlated. In accordance with this, the fluxes could rarely be re-fitted in case one or both of these parameters were set arbitrarily. This indicates that the parameters *ϕ*_3*r *_and *ϕ*_4*r *_have a certain significant correlation that must be fulfilled at termination to yield unique solutions for other fluxes. As expected, the flux estimates of TK1 and those of TA were found to be nonlinearly correlated with each other as depicted in Figure 5-B for *ν*_3*r *_and *ν*_4*r*_. The true solution, (*ϕ*_3*r*_, *ϕ*_4*r*_) = (0.6341, 0.4337) also lies on the curve. At the termination, it is obvious that the output sensitivities of ∂**x**_m_/∂*ϕ*_3*r *_and ∂**x**_m_/∂*ϕ*_4*r *_are low: the objective function reached a very small value of nearly zero (*f*($\hat{\Theta }$) < 1 × 10^-10^) and we get arbitrary but correlated values for *ϕ*_3*r *_and *ϕ*_4*r*_. Due to this, we cannot practically get *ϕ*_3*r *_and *ϕ*_4*r *_estimated correctly and, accordingly, *ν*_3_, *ν*_3*r*_, *ν*_4_, and *ν*_4*r*_, while the corresponding net fluxes can always be calculated from other correct flux estimates in terms of stoichiometry.

We observed that a unique estimation of all four fluxes would be possible only if the mass isotopomers of sedoheptulose 7-phosphate were additionally measured. It was observed that providing these mass isotopomers renders the output sensitivities of ∂**x**_m_/∂*ϕ*_3*r *_and ∂**x**_m_/∂*ϕ*_4*r *_much less dependent on starting points. In contrast, the output sensitivities vary strongly and even become zero when the mass isotopomers of sedoheptulose 7-phosphate are not involved in the objective.

Further to this, the fluxes were not found to be influenced by the glucose 6-phosphate isomerase fluxes represented by *ϕ*_2*r*_. Disregarding the random values of *ϕ*_2*r*_, the fluxes except *ν*_3_, *ν*_3*r*_, *ν*_4_, and *ν*_4*r *_converged to their true values. Also *ϕ*_3*r *_and *ϕ *_4*r *_did not show any correlation with *ϕ*_2*r *_as depicted in Figure 5-C and Figure 5-D, respectively. Hence, we can conclude that the observed nonlinear correlation between *ϕ*_3*r *_and *ϕ*_4*r *_becomes visible solely from using the different starting points for the optimization. In addition, including *ϕ*_2*r *_as an additional design parameter gives the same result for the fluxes, whereas *ϕ*_2*r *_results in randomly distributed values without any obvious pattern. This shows that the flux identification carried out by the model linearization and null space investigation is appropriate for its practical use.

## Discussion

The tradeoff between robustness and convergence speed is a central issue in numerical optimization [14]. The developed method fulfills both criteria and enables exact metabolic flux estimation. This was accomplished on the basis of parametrizing the metabolic flux network by compactification and hybridizing the merits of two different gradient-based algorithms. Interestingly, HATA, which hybridizes the worst local method with SQP ∇_user _and utilizes the tolerance adjustment strategy, has proven to be superior to other approaches. This may be by virtue of providing STRiN with a more appropriate starting point advanced by SQP. Thus, because STRiN is computationally inexpensive and rapid in the vicinity of solution, it achieves a fast convergence. Furthermore, the interactive switching between the algorithms prevents STRiN from accepting an unfavorable search direction without significant objective improvement.

Further to this, we observed that the parametrization by the compactification of independent [0, ∞)-fluxes into [0, 1)-variables is advantageous compared to the non-compactified case. When the optimization was performed by selecting the [0, ∞)-fluxes (*ν*_2*r*_, *ν*_3*r*_, *ν*_4*r*_, *ν*_5*r*_, *ν*_6*r*_, *ν*_7*r*_, *ν*_8*r*_, *ν*_14_, *ν*_14*r*_, *ν*_15*r*_, *ν*_16_, *ν*_17*r *_with upper bounds of 200) directly as design parameters, the convergence took drastically longer (27 ± 45 min) than the cases using the compactified fluxes. Moreover, optimization runs often failed to converge or terminated at suboptimal points. In comparison to this, introducing [0, 1)-fluxes improved both the robustness and speed of convergence as demonstrated above. The same advantage is probably achieved when using the [0, 1)-compactifications such as exchange fluxes or flux partitioning ratios applied elsewhere [9,24,35]. Our compactification approach allows a straight parametrization because independent fluxes can easily be recognized from the stoichiometric matrix and compactified using a single rule of (2). Moreover, it is straightforwardly differentiable when considering that compactified [0, 1)-fluxes are continuous and smooth in the [0, ∞)-flux space and *vice versa*.

The parameter scaling constant *α *introduced during the compactification provides a way to implement numerical flux estimation with efficiency. In particular, the parameter scaling constant *α *is updated during optimization to render the model's Jacobian matrix into better condition. This is advantageous for the gradient-based methods for computing the search direction because it typically involves the inverse of the Jacobian matrix as given by (6). We examined whether different *α *values affect the efficiency of optimization. The *α *value was observed to affect the convergence speed but not the accuracy of the optimization. When starting from the same initial guess applied to the optimization trial in Figure 4, the fastest convergence speed was obtained when *α *is fixed at 10 (about 200 s for *f*(**Θ**) < 10^-14^). The speed efficiency decreased if *α *is smaller or larger than 10. A trial with *α *adjustment initiated at *α *= 1 automatically updated *α *to 10 after a few sub-optimization trials and took about 300 s to achieve *f*(**Θ**) < 10^-14^. Although it was slower than when *α *was fixed at 10, the proposed strategy with *α *adjustment recognized the optimal value. Among the 200 HATA optimization runs with *α *adjusting between 1 and 10 initialized with *α *= 1, 112 cases updated *α *to 10 after a few to several sub-optimization trials while *α *remained 1 in 78 cases. Moreover, the time taken for the optimization runs using different starting points was not necessarily shorter when *α *was updated to 10. This indicates that the most adequate *α *value for the optimization may depend on starting conditions.

**Figure 6:**
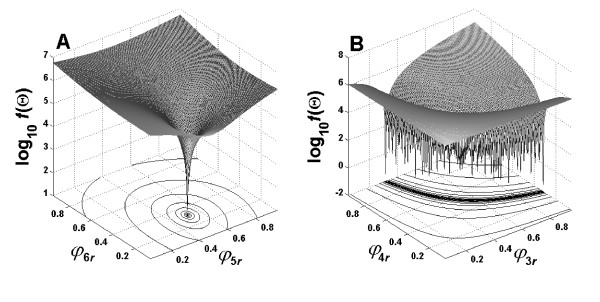
Behaviors of the objective function in the parameter space of non-correlated *ϕ*_5*r *_and *ϕ*_6*r *_(**A**) as well as correlated *ϕ*_3*r *_and *ϕ*_4*r *_(**B**). Two parameters were varied from 0.05 to 0.95 with a step of 0.005 while other parameters were fixed at the default solution **Θ**_default_.

We could find unique flux solutions except the fluxes recognized *a priori *as non-identifiable or those that were *a posteriori *found to have a significant nonlinear correlation. Such nonlinear parameter correlation can be identified only by the tedious process of executing flux estimation using different sets of starting values *a posteriori *[36]. The non-correlated [0, 1)-fluxes of which true values could be re-estimated, e.g., *ϕ*_5*r *_and *ϕ*_6*r*_, give a unique minimum of *f*(**Θ**), which is obviously achieved disregarding starting points (Figure 6-A). In comparison to this, the nonlinearly correlated [0, 1)-fluxes *ϕ*_3*r *_and *ϕ*_4*r *_result in an infinite number of minima that meet the correlation shown in Figure 5-B depending on starting points (Figure 6-B). Because the objective function value is nearly zero at each minimum (*f*($\hat{\Theta }$) < 1 × 10^-10 ^with a weighting factor of 10^8^), the system can be considered to have an infinite number of global minima when parameters are nonlinearly correlated. In this case, a global optimization method can be extremely expensive for identifying such unknown parameter correlations due to its time-inefficiency. As a consequence, a fast and reliable numerical method of flux estimation is highly desirable to repeat optimization runs at different starting points.

One interest of numerical optimization is whether the global solution can be obtained by the algorithm applied. We observed that the local minimizers from 200 random simulations agreed with each other and also with the true flux values, the global solution. Depending on experimental noise or metabolic state, the problem may contain a few to several local saddle points. In this case, one can consider a further hybridization with a global optimization approach. Global optimization algorithms are inefficient in terms of convergence speed. On the other hand, gradient-based algorithms can converge rapidly but lack a global perspective for non-convex problems. Also in other cases, the combination of global and local search procedures has proven to offer the advantages of both methods while offsetting their disadvantages [17,37-39]. Such hybridization with a global optimization algorithm is useful for non-convex problems containing several local minima and can be done in many ways [17,39,40]. For instance, the local minima obtained from a gradient-based method can be used to update the individuals in the population to prepare the next generation when combining with the GA [17,40,39,41]. Because we could prove the speed and accuracy of the developed optimization method, we consider it very useful for investigating metabolic fluxes and for developing efficient algorithms combined with a global optimization method.

In practice, the quality of the flux fitting can be judged by the *χ*^2^-test [42,43] when using noisy measurement data. In our case, the upper critical *χ*^2 ^limit is 206 at 95 % confidence level at the given degree of freedom of 174 (188 ^13^C labeling data + 5 effluxes - 19 design parameters). Hence, if the weighted objective value (1) is smaller than the critical value of 103 (this is due to the factor 1/2 in the objective function), the fit can be regarded as acceptable. This value can also be used as a termination criterion for the optimization control in Figure 1-E. As shown in Figure 4-A, the value was achieved at 150 s by HATA, at 170 s by SQP with ∇_user_, and at 750 s of optimization time by SQP with ∇_finite_. In comparison to this, GA or SA was expected to achieve the acceptable *f*(**Θ**) after 7-8 hours or more (by extrapolation of log_10 _*f*(**Θ**)with respect to optimization time). Hence, an efficient Monte Carlo simulation cannot be carried out by the global optimization approaches.

Further, we compared HATA with SQP with ∇_user _by conducting 100 stochastic simulations under identical conditions. At each simulation run, normal random numbers were generated for the experimental data set (^13^C mass isotopomer fractions and extracellular fluxes) by assuming a unit standard deviation of 1 × 10^-3 ^and uniform random numbers for the starting points of design parameters. Subsequently, parameter re-estimation was performed in terms of HATA and SQP with ∇_user_, respectively. Each optimization run was stopped if *f*(**Θ**) becomes smaller than the critical *χ*^2 ^limit of 103 or does not give any further improvement. On average, *f*(**Θ**) at termination was 102 ± 26 for HATA and 167 ± 567 for SQP with ∇_user_, and the time taken for the optimization was 353 ± 216 s for HATA and 1234 ± 1356 s for SQP with ∇ _user_. The results support that the developed hybrid algorithm using tolerance adjustment will be more efficient and robust when computing metabolic fluxes from noisy experimental data in a real-case application. In contrast, the efficiency of the SQP algorithm seems to be affected by initial conditions (starting points and experimental data set) when considering the large variation in *f*(**Θ**) as well as in the computation time. In this regard, HATA will also achieve the most efficient optimization in the practical sense whenever repeated optimization runs are necessary. A further reduction in computation time can be obtained by utilizing a minimal set of isotopomer balances described by Antoniewicz et al. (2007) [3].

## Conclusion

We developed and examined a developed hybrid algorithm for numerical ^13^C flux estimation using a metabolic network model parametrized using a single compactification rule. We could prove its practical usefulness using the central metabolic network of *Bacillus subtilis*, which is a prokaryotic model organism and an industrially relevant strain. Based on the parametrization by compactification and model identification, the hybrid algorithm with tolerance adjustment allowed a fast and robust flux computation from the ^13^C labeling data created from ^13^C-labeled succinate and glutamate feeding. Such fast optimization was also found to be essential for the *a posteriori *identification of possible parameter correlations and also for the Monte Carlo approach to obtain statistical qualities for metabolic flux estimates. Therefore, this work represents an important contribution to the quantitative study of metabolic networks in the framework of systems biology.

## Methods

For the flux re-estimation simulations of the central metabolic network of *B. subtilis*, the following inputs and outputs were applied.

### ^13^C Input Substrates

The choice of input tracer substrates was made by means of the *D*-optimality criterion [44]. Since parallel experiments using different ^13^C input labels yield more information as theoretically shown for the respirometric flux analysis utilizing CO_2 _labeling measurement [4]. Therefore, experimental designs for two parallel cultivations were also considered here. Among the investigated experimental designs (630 possible designs from commercially available ^13^C glutamate and ^13^C succinate species; refer to Additional file 1!), a design combining data from a ^13^C labeling experiment using non-labeled glutamate and [2,3-^13^C_2_] succinate with those from an experiment using [1,2-^13^C_2_] glutamate and [1,4-^13^C_2_] succinate was expected to provide the richest information at a reasonable expense. This design was selected to study flux identifiability and to examine the developed hybrid optimization.

### ^13^C Output Metabolites

For simulation studies, we assumed that the mass isotopomers of proteinogenic amino acids such as alanine, valine, serine, threonine, glycine, aspartate, isoleucine, leucine, phenylalanine, tyrosine, arginine, histidine, and glutamate are measurable. In addition, the mass isotopomers of ribose 5-phosphate from RNA hydrolysate and hexose from carbohydrate hydrolysate are also assumed to be measurable. This totally gives a mass isotopomer data size of 188 for the parallel experiment. The measurements of mass isotopomer distributions were assumed to have a unit standard deviation of 1 × 10^-4^.

### Software implementation

All simulations were carried out on a PC equipped with a CPU of 2.4 GHz. The hybrid optimization algorithm depicted in Figure 1 was implemented in MATLAB (The Mathworks Inc., Natick, MA). Numerical optimization was carried out using the Optimization Toolbox (Version 3.0.4) and the Genetic Algorithm and Direct Search Toolbox (Version 2.1) of MATLAB. The symbolic operations required for parametrization and for computing partial derivatives were conducted using the Symbolic Math Toolbox (Version 3.1.4) of MATLAB. During simulations, random numbers were generated using the Statistics Toolbox (Version 5.2) of MATLAB. The random numbers for [0, 1)-fluxes (*ϕ*_*i*_) were bounded such that the relative values of the corresponding dependent fluxes (**ν**_depend_) lie between 0 and 20.

An example of the hybrid optimization for the problem described in the current work was coded using MATLAB (Hybridoptimizer.m) and supplemented (see Additional file 2). The method can be incorporated with any isotopomeric models [2,3,5,8] that are analytically differentiable.

## List of abbreviations used

**BFGS**: Broyden-Fletcher-Goldfarb-Shanno; **GA**: genetic algorithm; **HATA**: hybrid algorithm with tolerance adjustment; **KEGG**: Kyoto Encyclopedia of Genes and Genomes; **MDV**: mass isotopomer distribution vector; **MFA**; metabolic flux analysis; **NLSP**: nonlinear least-squares problem; **SA**: simulated annealing; **SQP**: sequential quadratic programming **SQP∇**_**user**_: SQP optimization with analytical gradient; **SQP∇**_**finite**_: SQP optimization using the numerical gradient obtained by finite difference; **STRiN**: subspace trust-region method based on the interior-reflective Newton method; **STRiN∇**_**user**_**H**_**user**_: STRiN using analytical gradient and Hessian; **TA**: transaldolase; **TK**: transketolase

## Authors' contributions

TH Yang conceived the study, developed the concepts, implemented simulation, and drafted the manuscript. O. Frick prepared the data required for modeling of the stoichiometric and isotopomer network of *B. subtilis*. E. Heinzle supervised the work and was involved in writing the manuscript. All authors read and approved the final manuscript.

## Appendix

### Gradient and Hessian

At each *k*^th ^iteration of (1), the model function F is evaluated at the *k*^th ^iterate **Θ**_*k *_to get the values corresponding to **η **= (**ν**_m_, **x**_m_)^*T*^. This is done by generating a certain flux state by **ν**_*k *_= **F**_flux_(**Θ**_*k*_) and, subsequently, solving **F**_carbon_(**x**_*k*_, **ν**_*k*_) = **0 **for **x**_*k *_at the *k*^th ^flux state **ν**_*k*_. Since F is differentiable with respect to **Θ**_*k*_, the gradient ∇*f *can be computed analytically by:

$$\nabla f(\Theta_{k})=\frac{\partial F{\left(\Theta_{k}\right)}^{T}}{\partial \Theta }\cdot \Sigma_{\eta }^{-1}\cdot \left(F(\Theta_{k})-\eta \right).$$

Further to this, the Hessian matrix **H **that specifies the curvature of the search surface can be obtained by linearization [14,15], i.e.,

$$H_{k}=\frac{\partial F{\left(\Theta_{k}\right)}^{T}}{\partial \Theta }\cdot \Sigma_{\eta }^{-1}\cdot \frac{\partial F{\left(\Theta_{k}\right)}^{T}}{\partial \Theta }.$$

In practice, the constrained problem (1) is formulated as the Lagrangian function, a linear combination of the objective function and the constraints [14].

### Adjustment of tolerance and parameter scaling constant

The tolerance adjustment by the restart in Figure 1-E requires a series of sub-optimization trials. The first optimization trial starts with "sufficiently relaxed" tolerances both placed on parameters and objective function, e.g., of 100. When the *k*^th ^trial is acceptable (e.g., first order optimality conditions are satisfied to the specified tolerance or changes in parameters or function are smaller than the specified tolerance), the (*k *+ 1)^th ^trial restarts with a 10-fold decreased tolerance, where the *k*^th ^local minimizer Θ*_k _is used for the new starting point. The previous trial provides a more adequate starting point for the next, and it can be advantageous for local search methods. This is because the efficiency of local methods depends heavily on the quality of starting points [14,19]. In case the current trial fails (e.g., by exceeding the maximum number of iterations allowed in the estimation process), the tolerance is increased and the next trial starts from the iterate **Θ**° recorded for the smallest function value up to the current trial in the feasible region (Figure 1-E). The tolerance at which an optimization trial fails is registered. In case optimization trials successively fail, which is rarely the case, the step of tolerance increase is set smaller such as 5-, 10-, 50-, or 100-fold, etc., of the registered value. Correspondingly, the tolerance decrease is also made by this reduced step until the current tolerance matches the registered. This allows finding an appropriate tolerance value for NLSP.

At the beginning and at every failed trial, *α *is re-adjusted by referencing the condition number of the model's Jacobian matrix. When the new starting point for the *k*^th ^trial is **Θ**° that is isolated from the previous (*k *- *n*)^th ^trial with *α*_*k*-*n*_, the [0, 1)-fluxes have to be rescaled in accordance with the new scaling constant *α*_*k*_. Since *ν *= *α*_*k*-*n*_*ϕ*_*k*-*n*_/(1 - *ϕ*_*k*-*n*_) from (2), substituting *ν *in *ϕ*_*k *_= *ν*/(*α*_*k *_+ *ν*) results in:

$$\phi_{k}=\frac{\alpha_{k-n}\phi_{k-n}/(1-\phi_{k-n})}{\alpha_{k}+\alpha_{k-n}\phi_{k-n}/(1-\phi_{k-n})}.$$

### Deterministic Method of Feasible Starting Point Generation

When the random generation of starting points (see Figure 1-A!) is difficult, the feasible starting points can be found deterministically by minimizing a suitable objective. For instance, one can minimize the following objective:

$$\begin{array}{cccc} \underset{\Theta }{\min} & 2^{N_{\text{neg}\text{.flux}}}+\frac{1}{\left|\log10\sum_{i=1}^{n}\left|b_{\text{flux}}\right|\right|} & \text{subject to} & \{\begin{array}{c} 2^{N_{\text{neg}\text{.flux}}}=1\\ 0\leq \phi_{i}<1\\ \max(0,-\sqrt{\chi_{\text{1,}\varphi }^{\text{2}}}\sigma_{\text{efflux}})\leq \nu_{\text{efflux}}\leq \sqrt{\chi_{\text{1,}\varphi }^{\text{2}}}\sigma_{\text{efflux}} \end{array} \end{array}$$

Here, *N*_neg.flux _signifies the number of negative-valued fluxes, *b*_flux _the steady-state stoichiometric flux balances set up for metabolites (see the next section!) evaluated at the current iterate **Θ**_*k*_, and *n *the number of the balances. By the first term in the objective and the first constraint, the minimization is directed such that all fluxes become non-negative. The second term is to prevent flux values calculated that may not obey stoichiometry due to the limited floating-point accuracy (round-off error). This approach is useful for preparing initial values while preserving random nature for Monte Carlo simulations. In practice, the upper bound of *ϕ*_*i *_can be set to a value smaller than 1 that gives sufficiently large flux values so that *ϕ*_*i *_has a closed interval.

***Stationary State Stoichiometric Balances ***are set up around 15 metabolites specified in Figure 2 as follows. The values multiplied with Y_XS _equals the strain specific precursor demand for growth (mmol precursor per gram biomass).

glucose 6-P: *ν*_2 _- (*ν*_1 _+ *ν*_2*r *_+ 0.4217Y_XS_) = 0

fructose 6-P: (*ν*_2*r *_+ *ν*_4 _+ *ν*_5 _+ *ν*_6_) - (*ν*_2 _+ *ν*_4*r *_+ *ν*_5*r *_+ *ν*_6*r*_) = 0

glyceraldehyde 3-P: (*ν*_3 _+ *ν*_4*r *_+ *ν*_5 _+ 2*ν*_6*r *_+ *ν*_7_) - (*ν*_3*r *_+ *ν*_4 _+ *ν*_5*r *_+ 2*ν*_6 _+ *ν*_7*r *_+0.4659Y_XS_) = 0

3-phosphoglycerate: (*ν*_7*r *_+ *ν*_8_) - (*ν*_7 _+ *ν*_8*r *_+ 0.2209Y_XS_) = 0

phosphoenolpyruvate: (*ν*_16 _+ *ν*_8*r*_) - (*ν*_8 _+ *ν*_9 _+ 1.7854Y_XS_) = 0

pyruvate: (*ν*_9 _+ *ν*_15_) - (*ν*_10 _+ *ν*_15*r *_+ 0.9964Y_XS_) = 0

pentose 5-P: (*ν*_1 _+ 2*ν*_3*r *_+ *ν*_5*r*_) - (2*ν*_3 _+ *ν*_5 _+ 1.1622Y_XS_) = 0

erythrose 4-P: (*ν*_4 _+ *ν*_5*r*_) - (*ν*_4*r *_+ *ν*_5 _+ 0.4659Y_XS_) = 0

sedoheptulose 7-P: (*ν*_3 _+ *ν*_4*r*_) - (*ν*_3*r *_+ *ν*_4_) = 0

acetyl-CoA: *ν*_10 _- (*ν*_11 _+ acetate_ex _+ 3.1585Y_XS_) = 0

isocitrate: *ν*_11 _- *ν*_12 _= 0

*α*-ketoglutarate: (*ν*_12 _+ *ν*_17_) - (*ν*_13 _+ *ν*_17*r *_+ *α*-ketoglutarate_ex_) = 0

glutamate: (*ν*_17*r *_+ glutamate_ex_) - (*ν*_17 _+ 1.4769Y_XS_) = 0

succinate: (*ν*_13 _+ *ν*_14*r *_+ succinate_ex_) - (*ν*_14 _+ fumarate_ex_) = 0

oxaloacetate: (*ν*_14 _+ *ν*_15*r*_) - (*ν*_11 _+ *ν*_14*r *_+ *ν*_15 _+ *ν*_16 _+ 1.3131Y_XS_) = 0

## Supplementary Material

Additional file 1 Optimal Experimental Design. the relative information index expected from the tracer designs consisting of different ^13^C-glutamate and ^13^C-succniate for single and two parallel experiments. Click here for file

Additional file 2 Hybridoptimizer. an example of the hybrid optimization for the problem described in the current manuscript coded using MATLAB. Click here for file
