# Supplementary material for: Hybrid optimization for 13C metabolic flux analysis using systems parametrized by compactification
Source: BMC Syst Biol. 2008 Mar 26;2:29. doi: 10.1186/1752-0509-2-29 (PMC2333969; doi:10.1186/1752-0509-2-29)
Supplement: Additional file 1 — Optimal Experimental Design. the relative information index expected from the tracer designs consisting of different 13C-glutamate and 13C-succniate for single and two parallel experiments. [file 1752-0509-2-29-S1.doc]

**Optimal Experimental Design**

Computer-aided designs (CAD) can be used to optimize experimental conditions based on a particular optimality criterion. Among those, the so-called *D*-optimality criterion is a well-established method for multi-level experimental design concerning a regression model [1] and useful when accuracy of the parameters themselves is the primary concern. Details how to compute *D*-optimality values for a carbon labeling system are described elsewhere [2-4].

In common, to identify optimal design, *D*-values have to be numerically minimized. In case a reference experimental design is available, the quality of information expected from the new design can be evaluated by comparing it to the reference design. To this end, the relative information index$\bar{I}$ can be applied [2, 3].

$\bar{I}=\left(\frac{D_{\text{REF}}}{D_{\text{ND}}}\right)^{\frac{1}{2N_{\text{free}}}}$,

where *D*_REF_ denotes the *D*-value of the reference, *D*_ND_ that of the design to be compared and *N*_free_ the number of the independent variables in model. The larger the relative information index resulting from an experimental design, the more information can be predicted with respect to the input labeling pattern used for the reference experiment.

Here, we computed the relative information index expected from the tracer designs consisting of different ^13^C-glutamate and ^13^C-succniate for single (Table S. 1) and two parallel experiments (Figure S. 1). The maximum information is expected when combining an experiment using [3,4-^13^C_2_]glutamate and [2,3-^13^C_2_]succinate with an experiment with [U-^13^C_5_]glutamate and [1,2-^13^C_2_]succinate. This has 1.2-fold higher information compared to the design applied in the manuscript (parallel experiments using non-labeled glutamate + [2,3-^13^C_2_]succinate and [1,2-^13^C_2_]glutamate + [1,4-^13^C_2_]succinate), whereas its experimental cost is 1.7-fold higher.

**Table S. 1** All possible combinations from ^13^C-glutamate and ^13^C-succinate for a tracer experiment and expected relative information index and experimental cost.

| **list #.** | **labeling pattern** | | **relative information**  **index*** | **cost [$]**** |
| --- | --- | --- | --- | --- |
|  | **glutamate** | **succinate** |  |  |
| 1 | [1-^13^C_1_] | non-labeled | 0.05 | 44.27 |
| 2 | [2-^13^C_1_] | non-labeled | 0.46 | / |
| 3 | [3-^13^C_1_] | non-labeled | 0.35 | / |
| 4 | [4-^13^C_1_] | non-labeled | 0.35 | / |
| 5 | [5-^13^C_1_] | non-labeled | 0.46 | / |
| 6 | [1,2-^13^C_2_] | non-labeled | 0.50 | 105.89 |
| 7 | [3,4-^13^C_2_] | non-labeled | 0.44 | 105.00 |
| 8 | [U-^13^C_5_] | non-labeled | 0.02 | 96.37 |
| 9 | non-labeled | [1,4-^13^C_2_] | 0.52 | 16.63 |
| 10 | non-labeled | [2,3-^13^C_2_] | 0.72 | 20.94 |
| 11 | non-labeled | [U-^13^C_4_] | 0.02 | 71.46 |
| 12 | [1-^13^C_1_] | [1,4-^13^C_2_] | 0.54 | 60.90 |
| 13 | [1-^13^C_1_] | [2,3-^13^C_2_] | 0.68 | 65.21 |
| 14 | [1-^13^C_1_] | [U-^13^C_4_] | 0.02 | 115.73 |
| 15 | [2-^13^C_1_] | [1,4-^13^C_2_] | 0.61 | / |
| 16 | [2-^13^C_1_] | [2,3-^13^C_2_] | 0.54 | / |
| 17 | [2-^13^C_1_] | [U-^13^C_5_] | 0.51 | / |
| 18 | [3-^13^C_1_] | [1,4-^13^C_2_] | 0.47 | / |
| 19 | [3-^13^C_1_] | [2,3-^13^C_2_] | 0.75 | / |
| 20 | [3-^13^C_1_] | [U-^13^C_4_] | 0.53 | / |
| 21 | [4-^13^C_1_] | [1,4-^13^C_2_] | 0.47 | / |
| 22 | [4-^13^C_1_] | [2,3-^13^C_2_] | 0.75 | / |
| 23 | [4-^13^C_1_] | [U-^13^C_4_] | 0.53 | / |
| 24 | [5-^13^C_1_] | [1,4-^13^C_2_] | 0.61 | / |
| 25 | [5-^13^C_1_] | [2,3-^13^C_2_] | 0.54 | / |
| 26 | [5-^13^C_1_] | [U-^13^C_4_] | 0.51 | / |
| 27 | [1,2-^13^C_2_] | [1,4-^13^C_2_] | 0.65 | 122.52 |
| 28 | [1,2-^13^C_2_] | [2,3-^13^C_2_] | 0.49 | 126.84 |
| 29 | [1,2-^13^C_2_] | [U-^13^C_4_] | 0.47 | 177.35 |
| 30 | [3,4-^13^C_2_] | [1,4-^13^C_2_] | 0.28 | 121.63 |
| 31 | [3,4-^13^C_2_] | [2,3-^13^C_2_] | 0.75 | 125.95 |
| 32 | [3,4-^13^C_2_] | [U-^13^C_4_] | 0.66 | 176.46 |
| 33 | [U-^13^C_5_] | [1,4-^13^C_2_] | 0.70 | 113.00 |
| 34 | [U-^13^C_5_] | [2,3-^13^C_2_] | 0.57 | 117.31 |
| 35 | [U-^13^C_5_] | [U-^13^C_5_] | 0.02 | 167.83 |

* relative information index [2, 3] calculated using the design selected for the present study (parallel experiments using non-labeled glutamate + [2,3-^13^C_2_]succinate and [1,2-^13^C_2_]glutamate + [1,4-^13^C_2_]succinate) as reference.

** cost estimate for 7 ml culture medium containing 35 mM succinate and 45 mM glutamate as co-substrates.

**Figure S. 1.** Relative information index computed for two parallel experiments which are all possible combinations of experiments listed in Table S. 1 (list # corresponds to axis tick label). The maximum information (relative information index = 1) is expected when combining the experiment using [3,4-^13^C_2_]glutamate and [2,3-^13^C_2_]succinate (list # 31) with [U-^13^C_5_]glutamate and [1,2-^13^C_2_]succinate (list # 33). The relative information index for the design applied to the present study (circled) was 0.87.

1. Massart DL: **Handbook of chemometrics and qualimetrics**. Amsterdam ; New York: Elsevier; 1997.

2. Mollney M, Wiechert W, Kownatzki D, de Graaf AA: **Bidirectional reaction steps in metabolic networks: IV. Optimal design of isotopomer labeling experiments**. *Biotechnol Bioeng* 1999, **66**(2):86-103.

3. Arauzo-Bravo MJ, Shimizu K: **An improved method for statistical analysis of metabolic flux analysis using isotopomer mapping matrices with analytical expressions**. *J Biotechnol* 2003, **105**(1-2):117-133.

4. Yang TH, Heinzle E, Wittmann C: **Theoretical aspects of ^13^C metabolic flux analysis with sole quantification of carbon dioxide labeling**. *Comput Biol Chem* 2005, **29**(2):121-133.
